# Extended Family Outreach in Hereditary Cancer Using Web-Based Genealogy, Direct-to-Consumer Ancestry Genetics, and Social Media: Mixed Methods Process Evaluation of the ConnectMyVariant Intervention

**DOI:** 10.2196/43126

**Published:** 2023-04-20

**Authors:** Annie T Chen, Jennifer Huey, Sandra Coe, Jailanie Kaganovsky, Emily A Malouf, Heather D Evans, Jill Daker, Elizabeth Harper, Olivia Fordiani, Emma E Lowe, Caileigh McGraw Oldroyd, Ashlyn Price, Kristlynn Roth, Julie Stoddard, Jill N Crandell, Brian H Shirts

**Affiliations:** 1 Department of Biomedical Informatics and Medical Education University of Washington Seattle, WA United States; 2 Department of Laborabory Medicine and Pathology University of Washington Seattle, WA United States; 3 Center for Family History and Genealogy Department of History Brigham Young University Provo, UT United States; 4 Brotman Baty Institute for Precision Medicine Seattle, WA United States

**Keywords:** familial cancer, hereditary cancer, family history, pedigree building, cascade screening, distant relatives, breast cancer, BRCA1, BRCA2, partner and localizer of BRCA2, PALB2, Facebook, patient advocacy

## Abstract

**Background:**

Cascade screening, defined as helping at-risk relatives get targeted genetic testing of familial variants for dominant hereditary cancer syndromes, is a proven component of cancer prevention; however, its uptake is low. We developed and conducted a pilot study of the ConnectMyVariant intervention, in which participants received support to contact at-risk relatives that extended beyond first-degree relatives and encourage relatives to obtain genetic testing and connect with others having the same variant through email and social media. The support that participants received included listening to participants’ needs, assisting with documentary genealogy to find common ancestors, facilitating direct-to-consumer DNA testing and interpretation, and assisting with database searches.

**Objective:**

We aimed to assess intervention feasibility, motivations for participating, and engagement among ConnectMyVariant participants and their families.

**Methods:**

We used a mixed methods design including both quantitative and qualitative evaluation methods. First, we considered intervention feasibility by characterizing recruitment and retention using multiple recruitment mechanisms, including web-based advertising, dissemination of invitations with positive test results, provider recruitment, snowball sampling, and recruitment through web-based social networks and research studies. Second, we characterized participants’ motivations, concerns, and engagement through project documentation of participant engagement in outreach activities and qualitative analysis of participant communications. We used an inductive qualitative data analysis approach to analyze emails, free-text notes, and other communications generated with participants as part of the ConnectMyVariant intervention.

**Results:**

We identified 84 prospective participants using different recruitment mechanisms; 57 participants were ultimately enrolled in the study for varying lengths of time. With respect to motivations for engaging in the intervention, participants were most interested in activities relating to genealogy and communication with others who had their specific variants. Although there was a desire to find others with the same variant and prevent cancer, more participants expressed an interest in learning about their genealogy and family health history, with prevention in relatives considered a natural side effect of outreach. Concerns about participation included whether relatives would be open to communication, how to go about it, and whether others with a specific variant would be motivated to help find common ancestors. We observed that ConnectMyVariant participants engaged in 6 primary activities to identify and communicate with at-risk relatives: sharing family history, family member testing, direct-to-consumer genealogy genetic testing analysis, contacting (distant) relatives, documentary genealogy, and expanding variant groups or outreach. Participants who connected with others who had the same variant were more likely to engage with several extended family outreach activities.

**Conclusions:**

This study demonstrated that there is an interest in extended family outreach as a mechanism to improve cascade screening for hereditary cancer prevention. Additional research to systematically evaluate the outcomes of such outreach may be challenging but is warranted.

## Introduction

### Background

For many hereditary cancer-risk genes, guideline-endorsed screening can effectively identify cancer early and surgery can prevent cancer if a pathogenic variant is known [1]. A current challenge is identifying those who would benefit before they get cancer. One of the best methods is through cascade screening in families [2,3]. Cascade screening involves targeted genetic testing in relatives at risk of having a specific genetic variant. It is called cascade screening because testing can “cascade” from one person who tests positive to first-degree relatives and then to additional relatives of those who test positive [3,4]. This strategy has been shown to be cost-effective for *BRCA1*, *BRCA2*, and Lynch syndrome genes and is endorsed by national and international organizations [5-8]. However, cascade screening uptake in the United States is low, with only 10% to 30% of first- and second-degree relatives receiving genetic testing after hearing about the genetic results of a proband—the initial person identified in a family [3,9,10]. Barriers to cascade prevention relate to the structure of the health care system and the lack of effective patient education [3,7,11-17].

Extending cascade outreach beyond first-degree relatives has been proposed to identify nearly all individuals with hereditary cancer risk [2]. Two individuals with the same variant are likely to have a common ancestor [18]. Identifying this common ancestor can in turn lead to the identification of numerous *n*-degree relatives and opportunities for prevention through cascade testing. Traditionally, relatives who would benefit from cascade testing are identified through 3-generation pedigrees created by genetic professionals, and many studies have used this principle to connect families through rare disease mutations, creating very large multigenerational pedigrees [18-22]. However, there is a missed opportunity.

There are various tools that may be useful for finding distant relatives who share a variant and a common ancestor, and these are increasingly available on the web to the public. The use of direct-to-consumer (DTC) genetic testing in conjunction with social media for family history and relative finding is becoming increasingly common [23,24]. However, there can also be potential concerns with the use of these tools, including the need to ensure that those using these tools have the necessary information, support, and discussion to make informed decisions and process any feelings that may result from tool use [25].

Situations where clinicians have noted 2 patients who share the same rare variant and have identified previously unknown familial relationships have led to the identification of additional at-risk relatives (personal communication). However, this impromptu cancer prevention practice has not been implemented systematically as a public health activity. The potential benefits from applying web-based genealogy tools, DTC genealogy genetics results, and social media networks for cascade testing and hereditary cancer prevention have not been fully explored.

### Objectives

In this paper, we present a preliminary study of participant experiences with the ConnectMyVariant intervention, which aims to empower participants to engage in family outreach for cancer prevention. Intervention participants receive access to several services, including a central database of individuals interested in talking with others who had the same variant, guidance to participants on seeking and connecting with others with the same variant through web-based message boards hosted by patient advocacy groups and in social media forums, and assistance in the understanding of documentary genealogy and DTC ancestry testing platforms. In addition, a flexible plan was developed to listen to individual patient needs and respond to requests as they arose.

To characterize how participants identified and communicated with at-risk relatives, we performed a qualitative analysis of communication from the ConnectMyVariant intervention. We explored two main research questions: (1) What actions did ConnectMyVariant participants take to find and communicate risk information with their relatives? and (2) What motivations and concerns did participants have about their involvement in study activities? We concluded with implications and areas of need to improve services to connect individuals with the same variants.

## Methods

### Study Design

In this study, we used a mixed methods design, in which we sought to collect and analyze both quantitative and qualitative data and integrate these 2 forms of data in the analysis and presentation of results [26]. In part 1, we explored intervention feasibility by characterizing recruitment and retention using a combination of descriptive statistics and temporal visualization methods. In part 2, we characterized motivations, concerns, and engagement through project documentation of participant engagement in outreach activities and analysis of participants’ communications.

### Intervention

**Figure 1:**
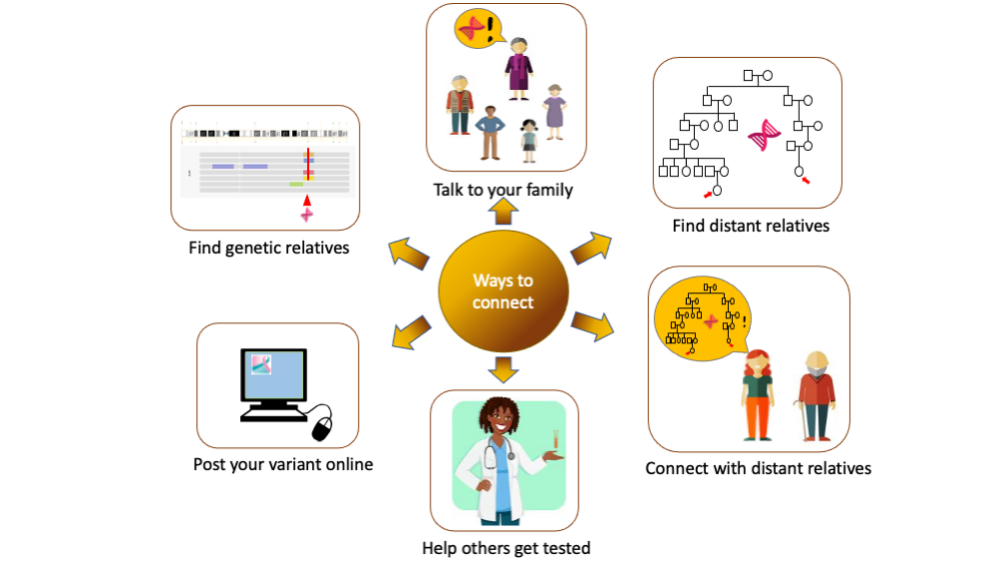
Ways to connect with others [27].

The ConnectMyVariant intervention provides educational information on how to spread awareness among families with regard to the risk of inherited diseases. The goal is to empower and assist families in finding others who may have their variant and share information about the disease risk that they might have. This can be done with close family members, distant relatives, relatives found through DNA ancestry testing, or on the web in discussion forums created to help people connect about variants (Figure 1 [27]).

Those who enrolled were sent a message (Multimedia Appendix 1) asking permission for the ConnectMyVariant intervention to share their contact information with others who had the same variant, encouraging them to find others with the same variant on social media, and suggesting that they seek common ancestors with others who share their variants. We created a publicly available website, ConnectMyVariant [28], with educational materials for the participants and their families (Figure 1). All family history and family communication activities were patient initiated and patient driven, with the research study team members making themselves available for guidance and advice whenever requested. All participants were offered their choice of AncestryDNA or MyHeritageDNA kits to help identify others who might be related. For those who used these kits, AncestryDNA or MyHeritageDNA accounts were created and owned by participants. DNA data were shared with the ConnectMyVariant team only if the participants chose to share information for specific genealogy-related purposes. Participants also had access to free, study-related genealogy assistance from the Brigham Young University Center for Family History and Genealogy (BYU CFHG). ConnectMyVariant leaders (BHS, JNC, and JS) worked together before the study to develop genealogy strategies that focus on helping people with hereditary cancer variants determine where in their family tree the variants came from, find common ancestors between ≥2 people with the same variant, and identify other at-risk individuals. This group met with genealogy researchers (HDE, JD, EH, OF, EEL, CO, AP, and KR) in biweekly meetings throughout the study to discuss progress and refine genealogy strategies.

### Ethics Approval and Participation

ConnectMyVariant began as an institutional review board–approved research study on August 1, 2019, and ended on January 11, 2021. The study procedures were approved by the University of Washington Institutional Review Board (00007349). Upon completion, the study was replaced with an ongoing public health initiative with the same name, goals, and activities. In mid-December 2020, each participant received an email asking if they would like to opt-in to participate in the public health initiative.

### Sample and Recruitment

Individuals could be eligible for the intervention in 2 ways: if they had received clinical testing that identified pathogenic or likely pathogenic hereditary cancer-risk variants or if they were relatives of individuals with hereditary cancer risk who did not have the variant themselves.

We recruited as many participants as possible between August 1, 2019, and January 11, 2021, using multiple recruitment mechanisms: (1) the intervention was featured on the Facing Our Risk of Cancer Empowered (FORCE) website; (2) it was advertised to patients receiving positive results from Ambry Genetics between June 5, 2020, and January 11, 2021; (3) patients found out about the intervention through word of mouth from genetics providers; (4) individuals heard about it in web-based forums from other participants; (5) ConnectMyVariant team members reached out to the providers of patients identified in the University of Washington Laboratory Medicine Database who had variants shared by others and asked them to contact their patients; and (6) ConnectMyVariant team members reached out to researchers who had published about the specific variants identified in other enrolled participants and asked them to contact those patients. If the ConnectMyVariant team communicated with a specific potential participant, the process data regarding contact and communication were included in the analysis. Participants who indicated that they were not interested in the intervention after hearing more about it were asked to describe their reasoning, if possible.

### Data Analysis

The first part of our analysis involved assessing intervention feasibility in terms of enrollment and retention. We calculated descriptive statistics for the sample, including representation of genes and variants among prospective participants and those who ultimately enrolled. Then, we characterized the participants’ engagement temporally in terms of the duration of study participation.

In the second part of our analysis, we considered motivations and concerns for participating and engagement with the intervention in terms of the activities performed, using both quantitative and qualitative evaluation methods. We performed qualitative data analysis using a general inductive approach, involving the preparation of the data, familiarization with the text, the creation of categories, and category refinement [29]. Our inductive analysis focused on communication between ConnectMyVariant staff and participants, including email and free-text notes. We exported these communications from REDCap (Research Electronic Data Capture; Vanderbilt University), the study database, and aggregated all communications by family. We then imported these data into the Dedoose qualitative data analysis software (SocioCultural Research Consultants) [30] and coded the data based on the actions that these families engaged in. One author (JH), a genetic counselor who is part of the ConnectMyVariant team, performed the initial coding. These codes were verified by a second author (BHS) in conjunction with discussion involving a third author (ATC). In the presentation of quotes illustrating themes, staff notes and participant email text was copy edited for clarity, and we provide information about the variants that participants had, as these variants may have shaped their experience and could potentially be of relevance in interpretation of the quote.

We performed triangulation of this analysis with records of whether participants engaged in the following activities: (1) connecting with the BYU CFHG for genealogy assistance, (2) using an AncestryDNA or MyHeritageDNA kit, and (3) posting information about their variant on the web through the FORCE Share Your Mutation message board, Facebook, or another web-based forum. We compared participation in these 3 activities among individuals who had been introduced to another participant who had the exact same genetic variant and those who did not share a variant with any other study participants using Fisher exact test to evaluate the significance of differences.

## Results

### Part 1: Recruitment and Retention

#### Recruitment

**Figure 2:**
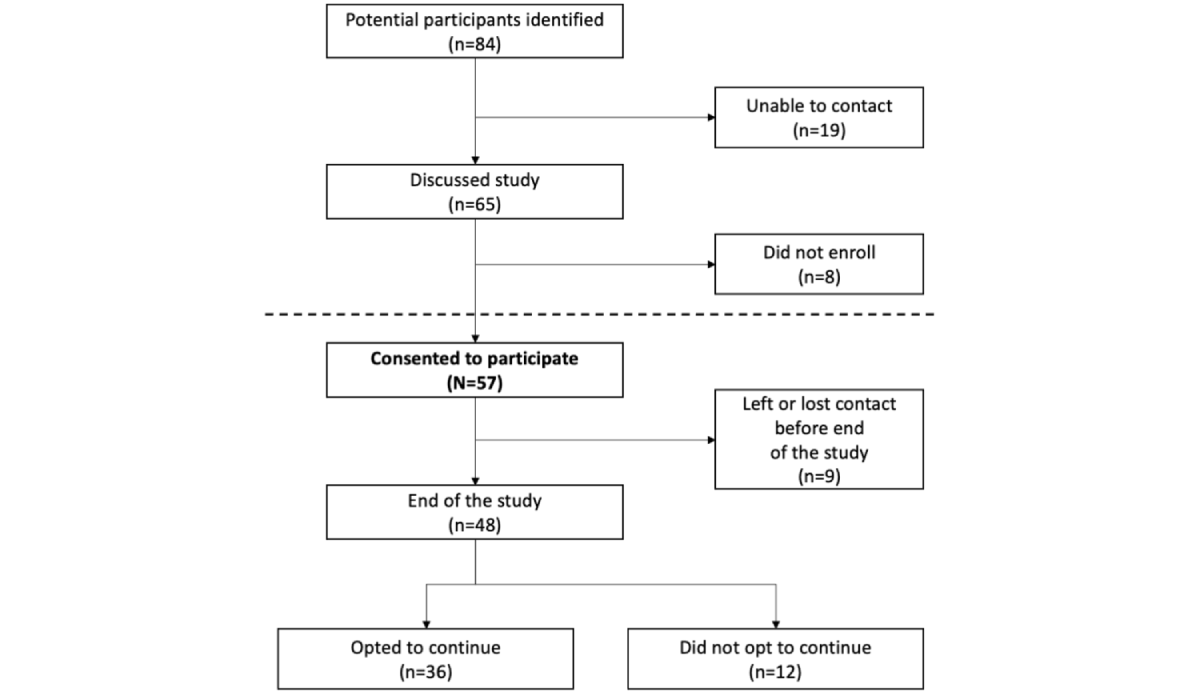
Diagram of intervention participation.

We identified 84 potential participants through the recruitment methods described in the *Methods* section. Figure 2 depicts our recruitment process, including the number of participants that we were able to contact, those who consented to participate, and those who ultimately transitioned into the public health initiative.

Table 1 depicts the extent to which we were able to contact and enroll the participants through these mechanisms (Table 1). The staff noted that 32% (27/84) of the participants learned about the study from FORCE and 11% (9/84) learned about it from Facebook. Of those who were identified by the study, 26% (22/84) were contacted through providers and 12% (10/84) were contacted through research studies. For 6% (5/84) of individuals, it was not clear if they found out about the study from FORCE, Facebook, other participants, or another source. Furthermore, 13% (11/84) of the prospective participants learned about the ConnectMyVariant intervention from other participants, suggesting that the snowball method may be a particularly promising form of recruitment.

**Table 1:** Contact and enrollment by recruitment method.

Recruitment method	Recruited (N=84), n (%)	Contacted			Enrolled	
		Participant (n=65), n	Recruited, n/N (%)	Participant (n=57), n		Recruited, n/N (%)
FORCE^a^	27 (32)	27	N/A^b^	26		26/27 (96)
Facebook	9 (11)	9	N/A	8		8/9 (89)
Other participants	11 (13)	10	10/11 (91)	8		8/11 (72)
Through providers	22 (26)	8	8/22 (36)	4		4/22 (18)
Research studies	10 (12)	6	6/10 (60)	6		6/10 (60)
Other or Unknown	5 (6)	5	N/A	5		5/5 (100)

^a^FORCE: Facing Our Risk of Cancer Empowered.

^b^N/A: not applicable. Individuals in these groups contacted the study directly to enroll rather than being contacted by the study.

Overall, 68% (57/84) of the individuals identified joined the study; however, those who self-identified (FORCE or Facebook) joined at a rate of 94% (34/36), whereas those who did not self-identify (found through other participants, medical records, or research studies) joined at a rate of 42% (18/43). There were extended conversations, involving multiple calls or emails over weeks or months between potential participants and the study team before participants decided whether to enroll.

Contacting individuals identified through the health care system was particularly challenging. The study staff members contacted providers and asked them to relay information to 22 patients; providers returned contact information so that staff could introduce the study to 8 (36%) patients and only 4 (18%) patients enrolled. Thus, of the 84 individuals identified as eligible, the study had direct contact with 65, of whom 57 enrolled. Data were not available on how many individuals were in the denominator of seeing the announcement about the ConnectMyVariant initiative on FORCE or Facebook.

#### Sample

Among the sample (n=57), 36 unique variants in 8 genes were represented. Table 2 lists the number of individuals per gene. The mean age of the participants was 50.5 (SD 14; range 28-76) years and were almost entirely women (54/57, 95%).

**Table 2:** Number of individuals by gene reported in the family.

Gene	Identified (N=84), n (%)	Enrolled (n=57), n (%)
*ATM*	1 (1)	1 (2)
*BRCA1*	26 (31)	20 (35)
*BRCA2*	32 (38)	19 (33)
*CHEK2*	2 (2)	2 (4)
*EPCAM*	1 (1)	1 (2)
*MSH2*	2 (2)	1 (2)
*MSH6*	2 (2)	0 (0)
*PALB2*	12 (14)	9 (16)
*RAD51C*	4 (5)	4 (7)
*TP53*	2 (2)	0 (0)

#### Participation Duration

**Figure 3:**
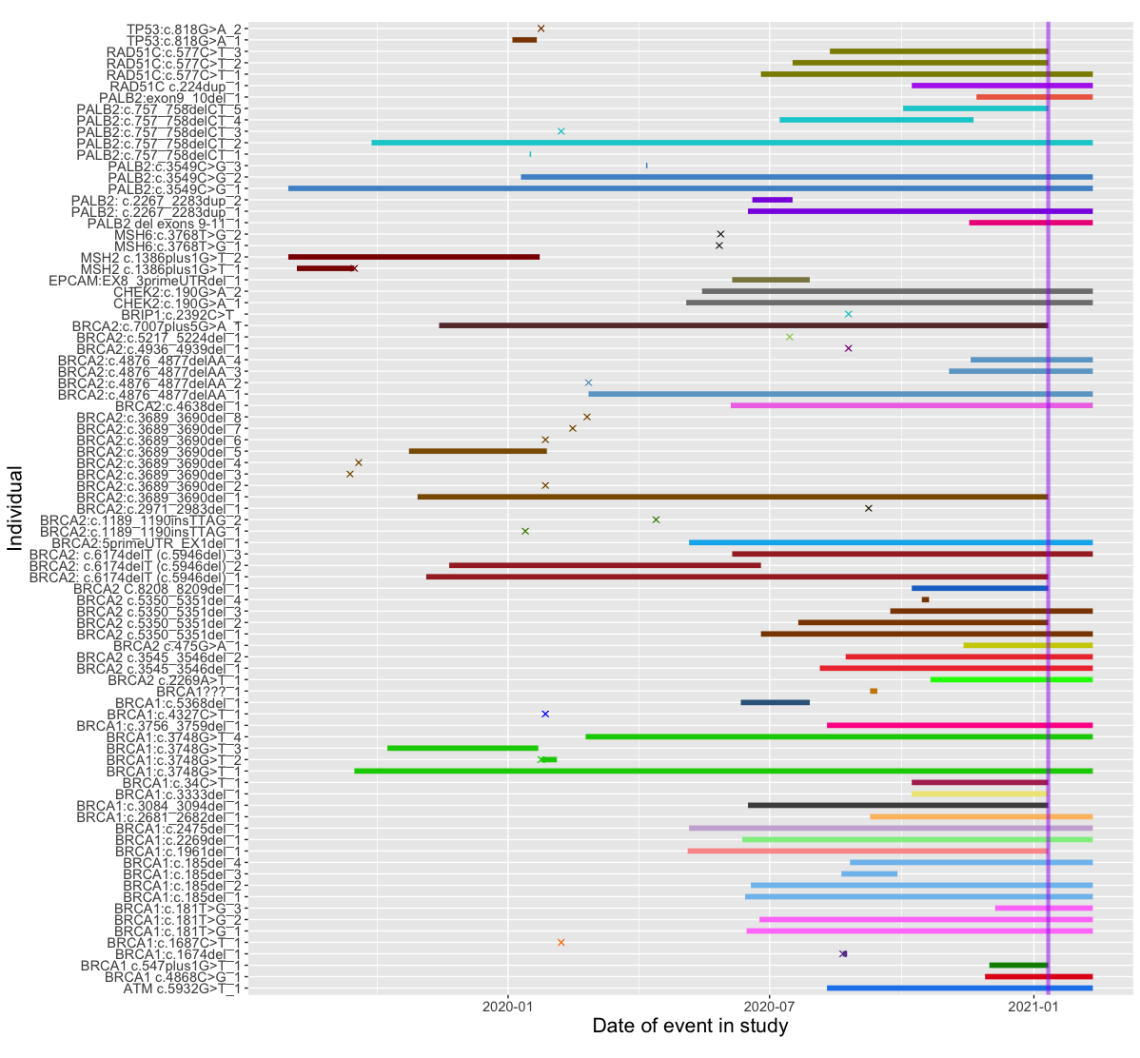
Duration of time with the connecting variant study after initial outreach. Time is shown along the x-axis starting at August 1, 2019. The end of the study is marked by the vertical line at January 11, 2021. Bars extending beyond the vertical line indicate participants who opted in to continue connecting with others through the ConnectMyVariant public health initiative. Groups of individuals sharing the same variant are plotted adjacent to each other using the same color. Bars for individuals who were identified but unable to be contacted or who did not enroll after one or more conversations are capped with “X” symbols.

The duration of participation in the project varied (Figure 3). When the study ended, 84% (48/57) of the participants were active, and 12% (7/57) of the participants had been active in the study for >1 year. Overall, 63% (36/57) of the participants chose to continue activities under the public health initiative after the study ended, and 21% (12/57) of the participants indicated that they did not want to engage further with the public health initiative. However, several of those who did not want to engage said they were still interested in being contacted by others with their variant, and 1 participant emailed the study team about their successful ongoing efforts to connect with distant relatives and help them get genetic testing.

### Part 2: Motivations, Concerns, and Engagement in ConnectMyVariant

#### Motivations and Concerns

We evaluated expressed motivations and concerns among the sample of 84 individuals identified as potential participants. Participants chose to enroll for various reasons. Some participants were interested in connecting with others:

I’m very interested in finding other distant relatives with the same mutation as me.

Participant BRCA1c2269del_1

Some participants wanted to help others or recognize the importance of the knowledge that they held:

I am on a sort of mission, to help spare lives from the same disease that has struck my family, because I was fortunate to benefit from genetic knowledge while my sisters were not. This is my way to “pay it forward” to the world.

Participant PALB2c757758delCT_2

But I do carry information that could save someone’s life. It probably saved mine (had all risk reducing surgery), although the decisions were brutal.

Participant BRCA1c547plus1GT_1

Some other participants were interested in learning more about the science:

I am very interested in learning as much as I can about this gene.

Participant PALB2c757758delCT_4

The participants also expressed their concerns. One common concern was whether family members would be open to the communication and how to go about it:

I think I would be okay with the conversation, although I’m not really sure how to just throw news like that out there either!

Participant BRCA1c2682del_1

I don’t want to make her feel I am being pushy.

Participant BRCA1c1961del_1

The intervention also raised questions and challenged us to find ways to help families in ways that suited them:

I am interested, however I find your study to be somewhat disorganized. That is concerning as I don’t want my information spread freely, but as I choose to find family members with my variant.

Participant BRCA1c3748GT_1

If she’s hesitant about fully participating in the project, but is interested in the genealogy side and trying to find a familial link with you, then we are happy to just connect her and you to the group of genealogists we’re partnering with at the BYU Center for Family History and Genealogy (CFHG).

ConnectMyVariant team members to participant BRCA1c3748GT_1

#### Activities

Through our qualitative data analysis, we observed that participants engaged in 6 main activities and how ConnectMyVariant team members supported those activities. These included sharing family history, family member testing, DTC genealogy genetic testing analysis, contacting (distant) relatives, documentary genealogy, and expanding variant groups or outreach.

### Sharing Family History With ConnectMyVariant Staff

Participants shared quite a bit of information with ConnectMyVariant team members about their family history. For example, the following participant shared both genetic information and a health history that she was aware for her family members as they understood it:

I do know who the carrier of my mutation was-my paternal grandmother. It’s unclear whether it came from her father or mother, but her father died young, possibly of cancer, so it may have been him. I do have a detailed history of my father’s mother’s siblings, who had any cancer (breast, ovarian, colon) and who their children were.

Participant BRCA1c547plus1GT_1

The ConnectMyVariant team would consider what had been shared with them and help participants decide on the next steps. For example, in the notes, the ConnectMyVariant team members noted having discussed:

We talked about the following: 1. Connecting with [participant] and seeing if the BYU group can help expand the family tree on both sides. Interested to see if there may be a connection between her maternal side with [participant’s] family simply due to the Russia tie...but I know that’s still a slim chance due to how common this mutation is. 2. Pursuing AncestryDNA data for her and her son (who has the same mutation). However, I did tell her that since this variant is so common, AncestryDNA may not be a super useful way to identify distant DNA relatives for the purposes of this study.

Participant BRCA2c6174delTc5946del_3

### Family Member Testing

One theme focused on family members getting tested. Participants reached out to at-risk relatives and encouraged them to get tested, and the ConnectMyVariant team offered assistance with how and what to communicate. One example of such communication is the following, in which a genetic counselor provides a template for a participant to reach out to a relative: “Attached is a Template for a ‘Family Letter’ that we hope is a very simple & neutral way to share information about your ATM variant with your biological relative. Hope this could help if you choose to reach out to him” (ConnectMyVariant team member, to participant ATMc5932GT_1). This theme appeared in the email communications of 30% (17/57) of families.

In some cases, participants were successful in their efforts to encourage relatives to get tested, and in other cases, they could not encourage them to get tested: “My three female cousins are trying to test since their dad won’t do it” (Participant PALB2c22672283dup_1). There are various reasons people might not get tested. For example, sometimes it was a matter of time:

Thank you for stepping in and getting things done for all of us. I am very interested but right now I am working 7 days a week...In December I will be off work and in [US state] visiting my Dad. I will be trying to get him to do the DNA test for me.

Participant BRCA2c48764877delAA_3

In one situation, a relative brought it up to their health care provider, who told her that it was unnecessary:

She said she did bring it up with her doctor after seeing the Facebook posts back during the span we were discussing her mom’s memorial services. Her doctor told her she doesn’t need to be tested and he wasn’t going to worry about it. I felt like with her risk be 50% of having it and she has six children, now grandchildren who are getting older, too, maybe she got bad information?

Participant BRCA1c1961del_1

The ConnectMyVariant team suggested that she talk to her relative and recommend seeking a genetic counselor for additional information. Team members also explained to participants that they understand it can be difficult to discuss these topics with their relatives, and they also suggested alternatives of how to communicate this information:

It can be uncomfortable to try to contact a relative (even a close relative) about a genetic test result that may impact their health. Even for those who regularly talk to their relatives, not everyone discusses the in-depth details of their medical care. To try to address this issue, we have drafted a letter that you can send to a relative as a good starting place for contact.

Participant BRCA1c1961del_1

### DTC Genetic Genealogy Testing Analysis

A substantial portion of families (38/57, 67%) mentioned the use of DTC genetic testing services such as AncestryDNA and MyHeritage to find their relatives. Participants sometimes sought relatives via GEDMatch, a third-party genealogy service that allows people to upload data from DTC genetic testing to find matches, and ConnectMyVariant team not only provided instruction to participants on how to perform searches but often also performed searches, shared the results with participants, and provided suggestions on what to do next:

I have attached an excel spreadsheet which includes individuals on GEDMatch that have segment matches with you at the location of your BRCA1 variant...it is unclear from which side of the family these individuals are related and it is also unclear if they have the BRCA1 variant. Thus, we encourage caution if you choose to reach out to them. I have attached a document which has some suggestions on how to make connections with individuals on ancestry testing sites who may be related.

ConnectMyVariant staff to participant BRCA1c185del_2

Participants also asked questions about how to interpret information that they find:

I’ve been emailing a woman who matched me on my BRCA1 variant, whom I found on GEDmatch using the search terms you gave me. Using the triangulation tool she looks to be an ancestor on my mother’s side...My query to you is what are the possible reasons that this woman matched me on the mutated section of my BRCA1 gene and has had ovarian cancer, yet doesn’t seem to have my variant.

Participant BRCA1c2269del_1

At times, the use of DTC genetic testing services could lead to potentially troubling knowledge:

I found out performing AncestryDNA testing on my great aunt. She was not related to my mother and I, both of whom had the same variant. This led me to dig deeper and reach out To DNAangels to help now search for my mother’s biological father. I know it was his side that passed down this gene. This is a complete shock to me. I have NOT told my mother yet and I have not had anyone I test based on finding out these results.

Participant BRCA2c3546del_1

### Contacting (Distant) Relatives

Participants often learned about people who they were related to by using the tools offered on GEDMatch, MyHeritage, and other databases. The ConnectMyVariant team provided information and guidance about how to contact relatives:

Most importantly, remember to respect your relative’s right to decide to follow up. Genetic risk can be hard for some people. Sometimes a relative may respond that they are not interested. Sometimes people are interested, but it is not a good time in their life. So just try to meet them where they are.

Participant BRCA1c2682del_1

Overall, 30% (17/57) of the families contacted cousins that had been identified as at risk through the intervention. The following excerpt illustrates personal guidance from the ConnectMyVariant team members about how a self-introduction to a distant relative might go:

I am contacting you because I believe we are distant cousins...we are probably 4th-5th cousins because we share three segments of DNA. I found you through my shared DNA matches in MyHeritage, looked at your family tree, did some Internet searching...and then through my Truthfinder subscription. I sent you a message on MyHeritage, but I also thought I’d try to reach out through email. There is a lot of breast cancer in my family. We found out that it is because of a specific genetic change in a cancer risk gene. I have been doing family history work to find others that have it.

Participant PALB2c757758delCT_2

The email threads showed that there were often multiple communications between the participants and the distant relatives whom they contacted. These communications showed it can take time for people to persuade distant relatives to get tested for various reasons, including having to work up the courage to contact them: “Just trying to still work up to feeling comfortable talking to him or texting” (Participant BRCA1c1961del_1). Sometimes, the participants’ inclinations to reach out were related to the probability of sharing a match:

Are you able to give me any sense of how likely it is that other people near the top of the list would have the BRCA1 mutation. I’m trying to decide whether I feel comfortable contacting them, and it would be good to know if it’s a fairly remote chance, or something that’s quite likely.

Participant BRCA1c185del_2

People are not always interested in getting tested or pursuing things further, which can lead to tension within the family. Participating reminded people of past experiences and led to recontacting those who had not been tested because of prior conversations:

I will probably follow up with my close cousin [name] (his mom has BRCA) and see where he is at with testing, but this will probably be my 4th time contacting him about it. There is a lack of interest for testing probably due to his own mom not pushing them to do it. She is also the one who found out she had the mutation back in 2012 when she was diagnosed with breast cancer for the second time and never told any family members. If she would have, my sister probably would not have gotten cancer, so we are slightly bitter about the lack of empathy and attention on her part.

Participant BRCA1c3084309del_1

### Documentary Genealogy

In total, 49% (28/57) of families attempted to expand their documented genealogy. Participants sometimes ran into difficulties in terms of the types of information sources that might be available, including challenges finding international records:

Would you be able to pass on any information regarding the origins of the mutation? I know it’s a Norwegian founding mutation, which makes sense since my descendents came over here from Norway. I read that it was due to a genetic drift after the bubonic plaque. I’m really interested in learning more about it, but haven’t found much info online. Do you know if Norway has their own database of BRCA variants?

Participant BRCA1c3084309del_1

The ConnectMyVariant team members would assist participants by providing information and introducing them to the genealogists at BYU CFHG:

I wanted to connect you with our partner genealogists at the Brigham Young University Center for Family History and Genealogy (BYU CFHG). Their role in the ConnectMyVariant project is to help expand your family tree and try to find connections with people you identify through online forums/message boards as well as through the DNA matches you’re searching.

ConnectMyVariant to participant BRCA1c2269del_1

Tracing genealogy could also lead to additional questions such as the following:

There are a couple cases in the [surname] family where an [surname] male married twice, after a first wife died. [Relative’s] line comes from children of the first wives, but if the mutation were found in children of the second wives, that would definitely prove the mutation came through the [surname] men...correct?

Participant PALB2c757758delCT_2

### Expanding Variant Group and Outreach

Participants also engaged in expanding variant groups and outreach activities. This is similar to the tracked variable of web-based outreach but is more expansive as it could also include outreach through other methods. Sometimes participants connected with one another via social media:

I was referred to you by [Person with variant]. We found each other through Facebook and share the same exact PALB2 genetic mutation.

Participant PALB2c2267228dup_2

I saw that you posted your mutation on FORCE and it looks like one other person has commented that they have your same variant!

ConnectMyVariant team members to participant BRCA2c4638del_1

In addition, the ConnectMyVariant team sometimes, but not always, was able to connect people with the same variant:

I am sending this email to formally connect you all simultaneously. You all have the same BRCA2c.5350_5351del and all indicated an interest in connecting with others who have your variant.

ConnectMyVariant team member to participants with BRCA2c.5350_5351del_1, BRCA2c.5350_5351del_2, and BRCA2c.5350_5351del_3

Presently, because we have no other participants with your variant in our project, the CFHG involvement will be limited.

ConnectMyVariant team member to participant RAD51Xx224dup_1

Participants were successful to varying degrees:

I’ve now had three relatives confirm they’ve found the mutation in their raw DNA. I’ve sent two of them an email to ask them what you suggested below so will wait to hear. I’ll also ask the third person. They’re all in different countries - UK, USA and Australia.

Participant BRCA1c2269del_1

[Participant’s relative] is the only other person I successfully made any progress with.

Participant PALB2c3549CG_1

### Connecting With Others With the Same Variant and Engagement

**Figure 4:**
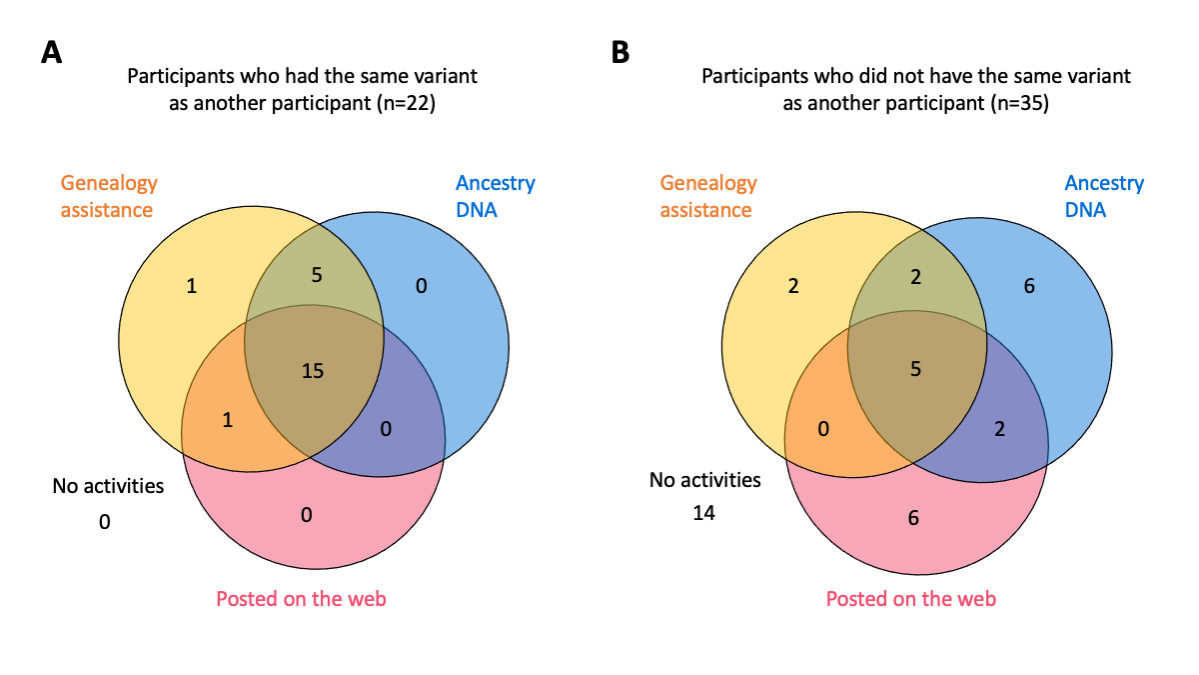
Connecting with others and engagement in study activities. (A) Participants connected with others who do share variants. (B) Participants who do not share variants with other study participants. Study activities: those who sought genealogy assistance from the Brigham Young University Center for Family History and Genealogy; those who requested or had already obtained AncestryDNA or MyHeritageDNA testing; and those who posted information about their variant on the web through the Facing Our Risk of Cancer Empowered Share Your Mutation message board, Facebook, or another web-based forum.

Among the 57 individuals who provided consent to participate in the study, 31 (54%) sought documentary genealogy assistance, 35 (61%) requested or had already undergone genealogy DNA testing, and 29 (51%) posted about their variant on at least 1 web-based forum (Figure 4).

We analyzed the study records to better understand the relationship between participant engagement in study activities and whether they were able to identify others with the same variant. Overall, 39% (22/57) of these participants shared variants with other ConnectMyVariant participants; all of these participants participated in at least 1 of 3 activities, and 26% (15/57) participated in all tracked activities (Figure 4A). Of the 35 participants who did not have the same variant as someone else in the study, only 5 (14%) participated in all 3 activities and 14 (40%) did not participate in any activities (Figure 4B). Connected participants were more likely to use genealogy assistance (*P*<.001), request Ancestry or MyHeritage DNA tests (*P*<.001), and post in web-based forums about their variant (*P*=.01). Individuals may have also been involved in other activities, such as communication with close and distant relatives, which were evaluated through qualitative analysis.

## Discussion

### Principal Findings

In the first part of our study, we identified potential participants through multiple recruitment mechanisms. Overall, 68% (57/84) of the potential participants enrolled in the study, with 84% (48/57) of the participants remaining engaged for the duration of the study. As enrollment was ongoing, participants engaged for varying lengths of time, but the study findings illustrate that it is possible to enroll and retain participants in cascade family outreach.

It is worth considering how our study recruitment might inform future cascade outreach efforts. In this study, the recruitment methods involving self-identifying mechanisms (eg, Facebook and FORCE) had higher yield. Although this alone might suggest that engaging those who are intrinsically motivated could be an effective strategy to raise awareness, it is also worthwhile to consider the particular dispositions of the sample, including the predominance of particular variants and all being women. In addition to pursuing high-yield avenues, there is also a need to increase efforts to diversify awareness and reduce barriers for persons who may benefit from cascade outreach but may have concerns about participating.

Participants experienced social and emotional challenges related to outreach to relatives or with the logistics related to identifying and communicating with relatives or availability of intervention resources. Some individuals chose not to participate despite knowing that someone with the same variant was interested in communicating with them, indicating that these activities do not appeal to everyone.

The ConnectMyVariant participants engaged in 6 primary cascade outreach activities: sharing family history, family member testing, DTC genealogy genetic testing analysis, contacting (distant) relatives, documentary genealogy, and expanding variant groups or outreach. Different families engaged in different activities and had varying strategies. Although some participants were compelled by a desire to find others and prevent cancer, more participants expressed an interest in finding out more about their family history and medical heritage, with prevention in relatives considered a natural side effect of outreach to distant relatives.

People who connected with others who had their variant were significantly more likely to participate in family history, genealogy DNA testing, and post on web-based forums about their genetic variant than those not connected with others. This observation is perhaps dialogic in the sense. One might expect that the more individuals there are seeking connections of a certain variant, the greater their chances of finding one another. However, there are also other factors, such as the amount of activity pertaining to a given variant on a discussion forum. A greater focus on forum management, communication, and dissemination of information via the web-based forums might increase the likelihood that individuals with the same variant would find one another.

The findings showed that ConnectMyVariant played an important role in facilitating discussion and sharing information. Some discussions were similar to those occurring in genealogy forums that cover technical topics such as shared DNA and how to find information in web-based genealogy databases (eg, Geneanet [31] and Ancestry message boards [32]), whereas others overlapped with those seen on hereditary cancer patient advocacy message boards, with comments on past cancer treatment experience and specific prevention plans (eg, FORCE message boards [33] and the American Cancer Society’s Cancer Survivors Network [34]).

However, there was a clear interest in using family history to identify connections among some persons at risk for genetic conditions. Common motivations included the desire to help others prevent cancer because of their own or their relative’s experience with cancer, a desire to understand their own personal genetics, or to improve science. Survey research has also shown that people connect with others via social media, particularly Facebook, in the context of rare genetic diagnoses [35]. In addition, research has shown that people use 23andMe results to make sense of their family and health histories, resolve unknowns about their pasts, make changes in day-to-day behaviors, and make sense of broader social and historical contexts [36]. Our study found that a substantial number of individuals with known hereditary cancer risk were interested in using social networking with documentary and genetic genealogy to build their family trees and identify new at-risk relatives. Engagement in these activities was enduring for approximately half of the enrolled participants. A few participants had independently started extended family outreach activities before the intervention began and welcomed ConnectMyVariant as a helpful resource that they had been hoping for. Interestingly, 53% (33/62) of the participants who began working with ConnectMyVariant opted to continue family connection and outreach efforts when the intervention transitioned to a public service (Figure 3). For some relatives, this project was a component of a multiyear, multiparticipant conversation embedded in deeper family communication related to cancer and mortality.

### Limitations and Future Directions

This study has various limitations. First, the ConnectMyVariant intervention was not a systematic study to gauge interest in cascade outreach among the general population; therefore, those enrolled are likely to overrepresent the level of interest among those who know about their hereditary cancer risk. In addition, given the complexity of facilitating this type of communication, our sample size was not insignificant, but there is a need to better understand how this approach to facilitate extended outreach might work in a larger and more diverse sample, including an analysis of different cultural groups.

Moreover, this study was not designed to assess the clinical outcomes related to genetic testing or prevention in relatives. Accurately measuring the clinical outcomes of extended family outreach is challenging owing to the heterogeneity of outcomes and the time frame of consequences. Each participant faced different family communication challenges and used different strategies to address these challenges. Moreover, the results of their actions may unfold over a time frame longer than is typically measured in a trial. For example, ConnectMyVariant occasionally receives emails from participants after their involvement with us has ended, informing us of something that they did that ultimately bore fruit, a year or more later. Additional work and new strategies will be required to monitor outcomes of expanding family outreach beyond first- and second-degree relatives and over an extended period.

### Conclusions

There is an interest and opportunity among individuals with hereditary cancer risk to extend cascade prevention beyond immediate relatives. In this paper, we presented an approach to facilitate this work. Social networking, documentary genealogy, and DTC genealogy testing can be leveraged to help while addressing limitations and concerns surrounding this use of technology.

## Appendix

Multimedia Appendix 1 Coding guide.

## Abbreviations

BYU CFHG: Brigham Young University Center for Family History and Genealogy
DTC: direct-to-consumer
FORCE: Facing Our Risk of Cancer Empowered
REDCap: Research Electronic Data Capture

## Data Availability

Deidentified data sets generated and analyzed during this study are available from the corresponding author upon reasonable request.
