# Supplementary material for: Extended Family Outreach in Hereditary Cancer Using Web-Based Genealogy, Direct-to-Consumer Ancestry Genetics, and Social Media: Mixed Methods Process Evaluation of the ConnectMyVariant Intervention
Source: JMIR Cancer. 2023 Apr 20;9:e43126. doi: 10.2196/43126 (PMC10160942; doi:10.2196/43126)
Supplement: Multimedia Appendix 1 [file cancer_v9i1e43126_app1.docx]

**Appendix: Coding Guide**

- Genealogy:
  - Noting when people were connected with BYU group
  - When people were sharing more distant family history information that they obtained through their own genealogy work
  - Updates from BYU group
- Contacting (distant) relatives:
  - If they mentioned contacting distant relatives based on their ancestry data analysis or the genealogy work by BYU
  - Re-contacting relatives whom they have spoken to about their variant, but it’s been a while
- Expanding variant group/outreach:
  - Outreach on FORCE, Facebook, etc
  - When we connect people together who have the same variant
  - When participants mention finding others with their same variant
- Family member testing:
  - If any of their relatives (immediate or distant) decide to pursue genetic testing
  - Or if relatives decline genetic testing
- DTC genetic testing analysis/comments:
  - Electing to pursue AncestryDNA, etc
  - Discussions of analyzing the ancestry DNA information/data
  - Information about what we found following GEDMatch analysis, etc and the identification of individuals who share segments of DNA and are likely (or not likely) to have the variant in question
    - We often made remarks such as “these are the individuals from your data who are at the highest risk of having your same variant”
- Participant concerns:
  - Self-explanatory
- Participant motivations:
  - Self-explanatory
- Sharing family history:
  - Initial family history information that is shared
    - Usually this was just 3-generation information
